# The KDM6A-KMT2D-p300 axis regulates susceptibility to diverse coronaviruses by mediating viral receptor expression

**DOI:** 10.1371/journal.ppat.1011351

**Published:** 2023-07-06

**Authors:** Jin Wei, Mia Madel Alfajaro, Wesley L. Cai, Vincent R. Graziano, Madison S. Strine, Renata B. Filler, Scott B. Biering, Sylvia A. Sarnik, Sonam Patel, Bridget L. Menasche, Susan R. Compton, Silvana Konermann, Patrick D. Hsu, Robert C. Orchard, Qin Yan, Craig B. Wilen

**Affiliations:** 1 Department of Laboratory Medicine, Yale School of Medicine, New Haven, Connecticut, United States of America; 2 Department of Immunobiology, Yale School of Medicine, New Haven, Connecticut, United States of America; 3 Hillman Cancer Center, University of Pittsburgh Medical Center, Pittsburgh, Pennsylvania, United States of America; 4 Department of Immunology, University of Connecticut Health Center, Farmington, Connecticut, United States of America; 5 Division of Infectious Diseases and Vaccinology, School of Public Health, University of California, Berkeley, Berkeley, California, United States of America; 6 University of Colorado Boulder, Boulder, Colorado, United States of America; 7 Department of Comparative Medicine, Yale School of Medicine, New Haven, Connecticut, United States of America; 8 Department of Biochemistry, Stanford University School of Medicine, Stanford, California, USA; 9 Arc Institute, Palo Alto, California, United States of America; 10 Department of Bioengineering, University of California, Berkeley, Berkeley, California, United States of America; 11 Innovative Genomics Institute, University of California, Berkeley, Berkeley, California, United States of America; 12 Center for Computational Biology, University of California, Berkeley, California, United States of America; 13 Department of Immunology, University of Texas Southwestern Medical Center, Dallas, Texas, United States of America; 14 Department of Pathology, Yale School of Medicine, New Haven, Connecticut, United States of America; 15 Yale Cancer Center, Yale School of Medicine, New Haven, Connecticut, United States of America; The Ohio State University, UNITED STATES

## Abstract

Identification of host determinants of coronavirus infection informs mechanisms of pathogenesis and may provide novel therapeutic targets. Here, we demonstrate that the histone demethylase KDM6A promotes infection of diverse coronaviruses, including SARS-CoV, SARS-CoV-2, MERS-CoV and mouse hepatitis virus (MHV) in a demethylase activity-independent manner. Mechanistic studies reveal that KDM6A promotes viral entry by regulating expression of multiple coronavirus receptors, including *ACE2*, *DPP4* and *Ceacam1*. Importantly, the TPR domain of KDM6A is required for recruitment of the histone methyltransferase KMT2D and histone deacetylase p300. Together this KDM6A-KMT2D-p300 complex localizes to the proximal and distal enhancers of *ACE2* and regulates receptor expression. Notably, small molecule inhibition of p300 catalytic activity abrogates ACE2 and DPP4 expression and confers resistance to all major SARS-CoV-2 variants and MERS-CoV in primary human airway and intestinal epithelial cells. These data highlight the role for KDM6A-KMT2D-p300 complex activities in conferring diverse coronaviruses susceptibility and reveal a potential pan-coronavirus therapeutic target to combat current and emerging coronaviruses.

**One Sentence Summary:** The KDM6A/KMT2D/EP300 axis promotes expression of multiple viral receptors and represents a potential drug target for diverse coronaviruses.

## Author summary

Three highly pathogenic coronaviruses SARS-CoV, MERS-CoV, and SARS-CoV-2 have spilled over into the human population in the last two decades. It is important to determine the mechanisms underlying how these viruses cause infection in order to increase our understanding of how viruses cause disease and to aid in the development of new therapeutics against current and future coronaviruses. Here we identify that the host proteins KDM6A, KMT2D, and p300 are critical for coronavirus infection. KDM6A, KMT2D, and p300 are important proteins implicated in cancer and developmental syndromes. They modify histone proteins, which regulate expression of specific host genes. We show that the KDM6A-KMT2D-p300 axis turns on expression of the host genes ACE2 and DPP4, which encode the receptors for SARS-like coronaviruses and MERS-CoV, respectively. KDM6A, KMT2D, and p300 act on the enhancer regions of the coronavirus receptor genes to turn on receptor expression. Small molecule inhibitors of p300 activity block receptor expression in both cell lines and primary human airway cells. This inhibition is well tolerated and makes the cells resistant to infection. This highlights the potential for targeting the KDM6A-KMT2D-p300 axis for diverse coronavirus infections.

## Introduction

Severe acute respiratory syndrome coronavirus 2 (SARS-CoV-2), the causative agent of COVID-19, has triggered an ongoing global pandemic. Highly effective COVID-19 vaccines have been approved and widely distributed; however, emerging SARS-CoV-2 variants highlight the need for novel anti-viral strategies against current and emerging coronaviruses [1]. Enhanced understanding of virus-host interactions at the cellular and molecular levels is critical for the development of both prophylactic and therapeutic approaches [2]. Currently authorized direct acting antivirals target the viral polymerase (remdesivir and molnupiravir) and viral protease (nirmatrelvir). All anti-virals are likely to select for drug-resistance at some level and thus new drug classes with broad activity are needed against currently circulating and pre-emergent coronaviruses [3–7]. Host-directed therapeutics provide a particularly promising approach given the potentially higher barrier to drug resistance, increased breadth of activity across coronaviruses variants and species, and the likelihood of synergy with direct-acting anti-viral drugs [8–10].

Coronavirus entry is mediated by the interaction of the viral spike (S) glycoprotein with a cellular receptor. Seven human coronaviruses have been identified, including four circulating seasonal coronaviruses (HCoV-229E, HCoV-OC43, HCoV-NL-63, HCoV-HKU1) and three highly pathogenic zoonotic coronaviruses (SARS-CoV, SARS-CoV-2 and Middle East Respiratory Syndrome-coronavirus (MERS-CoV)) [11–13]. Three of the seven human coronaviruses: SARS-CoV and SARS-CoV-2, and HCoV-NL-63 use ACE2 as a receptor, whereas MERS-CoV and HCoV-229E use DPP4 and APN as receptors, respectively [14–17]. Mouse hepatitis virus (MHV), a prototypic betacoronavirus, uses mouse Ceacam1 as a receptor [18]. After receptor engagement spike requires proteolytic processing, which can be mediated by several proteases including the transmembrane protease TMPRSS2 and the endosomal protease Cathepsin L [19–22]. Upon viral entry, viral RNA is released into the cytoplasm where it is translated and establishes viral replication and transcription complexes before assembling and budding [23–25].

We recently performed a genome-wide CRISPR screen to identify host genes essential for highly pathogenic coronavirus infection in African green monkey Vero E6 cells [26]. This genetic screen identified epigenetic regulators, including *KDM6A*, *KMT2D* and *EP300*, as top pro-viral genes for highly pathogenic coronavirus infection [26]. These genes have also been identified in other CRISPR screens for SARS-CoV-2 infection performed in human cell lines [27–30]. Lysine demethylase KDM6A (also known as UTX) belongs to the KDM6 family of histone H3 lysine 27 (H3K27) demethylases, which plays critical roles in homeostatic gene expression, cell differentiation, development and cancer [31–35]. KDM6A physically and functionally interacts with transcription factors (TFs), chromatin-modifying complexes, and histone modifying enzymes including H3K4 methyltransferases KMT2C/KMT2D (also known as MLL3/MLL4) and H3H27 acetyltransferases CBP/p300 [35–40]. KDM6A and KMT2D are frequently mutated in Kabuki syndrome and multiple cancers [41–43]. KMT2C/KMT2D and p300 are the key histone writers for establishing the active enhancer landscape by depositing H3K4me1 and H3K27ac at the active enhancer regions [44–46]. Recent studies indicate that KMD6A recruits KMT2D and p300 to mediate active enhancer landscape in a demethylase activity-independent manner [32,39]. However, the mechanisms by which KDM6A, KMT2D, and p300 mediate diverse coronavirus infection remains unknown.

Here, we demonstrate that the KDM6A-KMT2D-p300 axis is required for diverse coronavirus infection and viral entry in multiple cell lines and primary human airway epithelial cells. We show that KDM6A is essential for expression of ACE2, DPP4 and Ceacam1 in a demethylase-independent manner across multiple cell types from multiple species. Genetic inactivation of KMT2D or p300 blocked diverse coronavirus infection and viral receptor expression. Mechanistically, KDM6A recruits KMT2D and p300 through its TPR domain to regulate viral receptor expression and viral entry. Loss of KMT2D impairs the recruitment of p300 to KDM6A. Furthermore, knockout (KO) of KDM6A blocks the occupancy of KMT2D and p300 at *ACE2* proximal and distal enhancers. Finally, inhibition of p300 acetyltransferase activity using a selective and potent small molecule inhibitor attenuates receptor expression and reduces diverse coronavirus infection. Importantly, inhibition of p300 inhibits all major SARS-CoV-2 variants of concern (VOCs) and MERS-CoV infection in primary human airway and intestinal epithelial cells. Together, these data indicate KDM6A-KMT2D-p300 axis as a critical regulator of infection by diverse coronaviruses and represents a potential host-directed prophylactic and therapeutic target.

## Results

### KDM6A is required for highly pathogenic coronavirus infection

**Fig 1:**
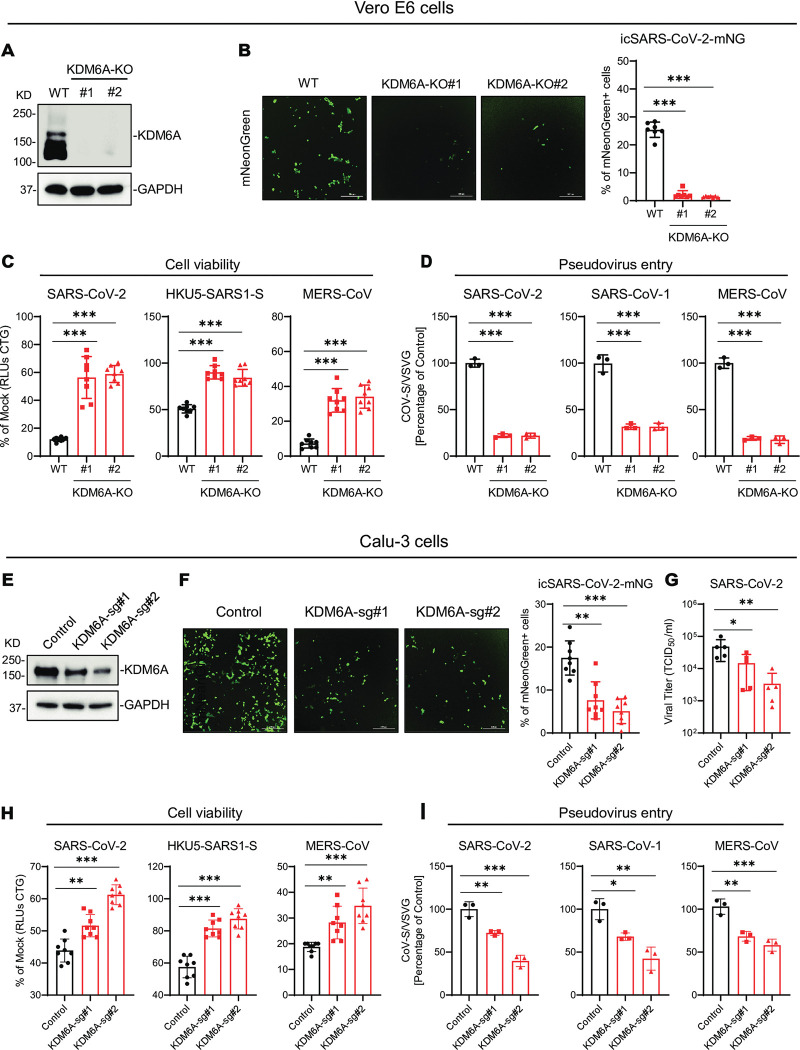
KDM6A is required for highly pathogenic coronavirus infection. **(A)** CRISPR-mediated KDM6A knockout clones were confirmed by Western blot in Vero E6 cells. **(B)** KDM6A KO cells were infected with icSARS-CoV-2-mNG at an MOI of 1. Infected cells were imaged via fluorescence microscopy (left) and mNeonGreen expressing cell frequency was measured 2 dpi (right). Scale bar: 300 μm. **(C)** KDM6A KO Vero E6 cells were infected with SARS-CoV-2 (left), HKU5-SARS-CoV-1-S (middle) and MERS-CoV (right) at an MOI of 0.2. Cell viability relative to a mock infected control was measured 3 dpi with CellTiter Glo. **(D)** KDM6A KO Vero E6 cells were infected with VSV peudovirus (VSVpp): VSV-G, SARS-CoV-2-S (left), SARS-CoV-1-S (middle), and MERS-CoV-S (right). Luciferase relative to the VSVpp-VSV-G control was measured 1 dpi. **(E)** KDM6A expression in KDM6A polyclonal KO Calu-3 cells. **(F)** KDM6A polyclonal KO Calu-3 cells were infected with icSARS-CoV-2-mNG at an MOI of 1. Infected cells were imaged via fluorescence microscopy (left) and mNeonGreen expressing cell frequency was measured 2 dpi (right). Scale bar: 300 μm. **(G)** Calu-3 cells were infected with SARS-CoV-2 at an MOI of 0.1 for 1 dpi. Virus titer was measured by TCID50. **(H)** KDM6A KO Calu-3 cells were infected with SARS-CoV-2 (left), HKU5-SARS-CoV-1-S (middle) and MERS-CoV (right) at an MOI of 0.2. Cell viability relative to a mock infected control was measured 3 dpi with CellTiter Glo. **(I)** KDM6A KO Calu-3 cells were infected with VSV peudovirus (VSVpp): VSV-G, SARS-CoV-2-S (left), SARS-CoV-1-S (middle), and MERS-CoV-S (right). Luciferase relative to the VSVpp-VSV-G control was measured 1 dpi. Data were analyzed by one-way ANOVA with Tukey’s multiple comparison test. Shown are mean ± SEM. *p < 0.05, **p < 0.01, ***p < 0.001.

Our previous genetic screens identified KDM6A as a potential pan coronavirus host factor [26]. To investigate the pro-viral role of KDM6A, we generated two independent single-cell KDM6A KO clones in Vero E6 cells using CRISPR/Cas9 and confirmed knockout efficiency by western blot (**Fig 1A**). We challenged cells with a replication-competent infectious clone of SARS-CoV-2 expressing the fluorescent reporter mNeonGreen (icSARS-CoV-2-mNG) and quantified the frequency of infected cells by microscopy [47]. Genetic inactivation of KDM6A blocked icSARS-CoV-2-mNG replication compared to WT cells (**Fig 1B**). Consistent with this, inactivation of KDM6A reduced the frequency of SARS-CoV-2, a bat coronavirus HKU5 expressing SARS-CoV spike (HKU5-SARS1-S), and MERS-CoV induced cell death (**Fig 1C**). Finally, we used pseudovirus assays to determine whether KDM6A regulates viral infection at the level of entry. Again, inactivation of KDM6A inhibited SARS-CoV, SARS-CoV-2 and MERS-CoV pseudovirus entry (**Fig 1D**). Collectively, these data demonstrate that KDM6A is required for highly pathogenic coronavirus entry into cells.

To assess whether the pro-viral role of KDM6A is restricted to Vero E6 cells, we generated polyclonal KDM6A KO cells in two human cell lines Calu-3 and Huh7.5, derived from human lung and liver, respectively. Calu-3 and Huh7.5 cells endogenously express ACE2 and DPP4 and support SARS-CoV-2 and MERS-CoV infection. Disruption of KDM6A conferred protection from SARS-CoV-2-induced cell death, reduced SARS-CoV-2 replication and pseudovirus entry in Calu-3 cells and Huh7.5 cells (**Figs 1E**–**1H and S1A**–**1D**). These findings demonstrate a conserved role for KDM6A across cell types and primate species.

### KDM6A regulates ACE2 and DPP4 expression in a demethylase-independent manner

**Fig 2:**
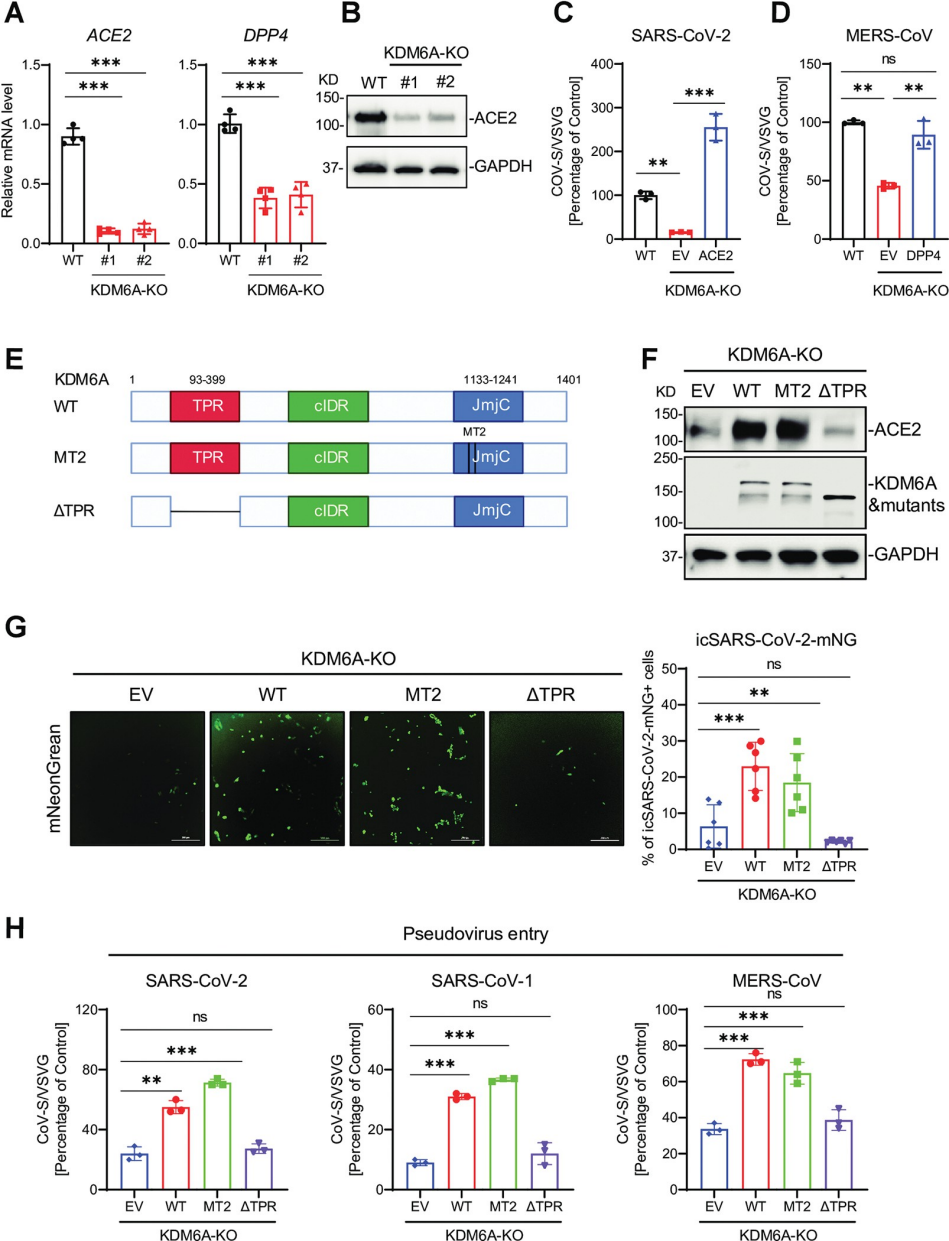
KDM6A regulates ACE2 and DPP4 expression in a demethylase-independent manner. **(A-B)** ACE2 and DPP4 expression in KDM6A KO Vero E6 cells at mRNA level (A) and protein level (B). **(C-D)** VSVpp- based pseudovirus entry in WT Vero E6 cells and KDM6A KO cells rescued with either human ACE2 (C) or human DPP4 (D). **(E)** Schematic of full length KDM6A and its mutants. **(F)** Immunoblot for ACE2 and KDM6A in KDM6A rescued cells. **(G)** KDM6A KO rescued cells were infected with icSARS-CoV-2-mNG at an MOI of 1. Infected cells were imaged via fluorescence microscopy (left) and mNeonGreen expressing cell frequency was measured 2 dpi (right). Scale bar: 300 μm. **(H)** KDM6A KO rescued Vero E6 cells were infected with VSV pseudovirus (VSVpp): VSV-G, SARS-CoV-2-S (left), SARS-CoV-S (middle), and MERS-CoV-S (right). Luciferase relative to the VSVpp-VSV-G control was measured 1 dpi. Data were analyzed by one-way ANOVA with Tukey’s multiple comparison test. Shown are mean ± SEM. *p < 0.05, **p < 0.01, ***p < 0.001.

To investigate how KDM6A regulates viral entry, we next assessed viral receptor expression. Inactivation of KDM6A reduced ACE2 and DPP4 mRNA levels as measured by qPCR and ACE2 protein levels as measured by western blot. (**Fig 2A and 2B**). DPP4 protein is below the limit of detection in wild-type cells and thus it could not be assessed in KO cells. We next performed RNA sequencing in KDM6A polyclonal KO Vero E6 cells. We found that ACE2 was one of the most down-regulated genes in KDM6A disrupted cells compared to control cells **(S2A Fig)**. KEGG pathway analysis revealed that several pathways are enriched amongst deferentially expressed genes in both directions, including ECM-receptor interaction, fatty acid biosynthesis, HIF-1 signaling pathway **(S2B Fig)**. Given that *ACE2* and *DPP4* are downregulated upon KDM6A deletion, we sought to determine if viral infectivity could be rescued in KDM6A KO cells following lentiviral transduction of recombinant human ACE2 and DPP4 **(S2C and S2D Fig)**. Indeed, lentiviral expression of ACE2 and DPP4 in KDM6A KO Vero E6 cells rescued SARS-CoV-2 and MERS-CoV infectivity, respectively, as assessed by pseudovirus infection (**Fig 2C and 2D**), whereas expression of ACE2 didn’t rescue MERS-CoV infectivity and vice versa (**S2E and S2F Fig**). This demonstrates that KDM6A-mediated regulation of viral receptor expression (ACE2 and DPP4) is responsible for the pro-coronavirus phenotype observed.

KDM6A is a histone H3K27 demethylase which demethylates di-methylated as well as tri-methylated histone H3 Lys27 (H3K27me3) [31]. However, KDM6A mediates tumor suppression and developmental regulation through noncatalytic functions [32,35,36]. To determine whether the catalytic activity of KDM6A is required for its pro-viral activity, we reintroduced the wild-type KDM6A (WT), a catalytically dead KDM6A mutant (MT2) [32,39], lacking a tetratricopeptide repeat (TPR) (ΔTPR) mutant or empty vector (EV) in KDM6A KO Vero E6 cells and confirmed expression by western blot (**Fig 2E and 2F**). Importantly, reintroduction of wild-type KDM6A (WT) and MT2 mutant, but not the ΔTPR mutant rescued ACE2 expression in KO cells (**Fig 2F**). Consistent with this, reintroduction of KDM6A WT and MT2, but not ΔTPR restored the icSARS-CoV-2-mNG replication and pseudovirus infectivity (**Fig 2G**–**2H**). These data demonstrate that KDM6A promotes highly pathogenic coronavirus infection in a demethylase-independent and TPR domain-dependent manner.

### KDM6A recruits KMT2D and p300 through the TPR domain for receptor expression and viral entry

**Fig 3:**
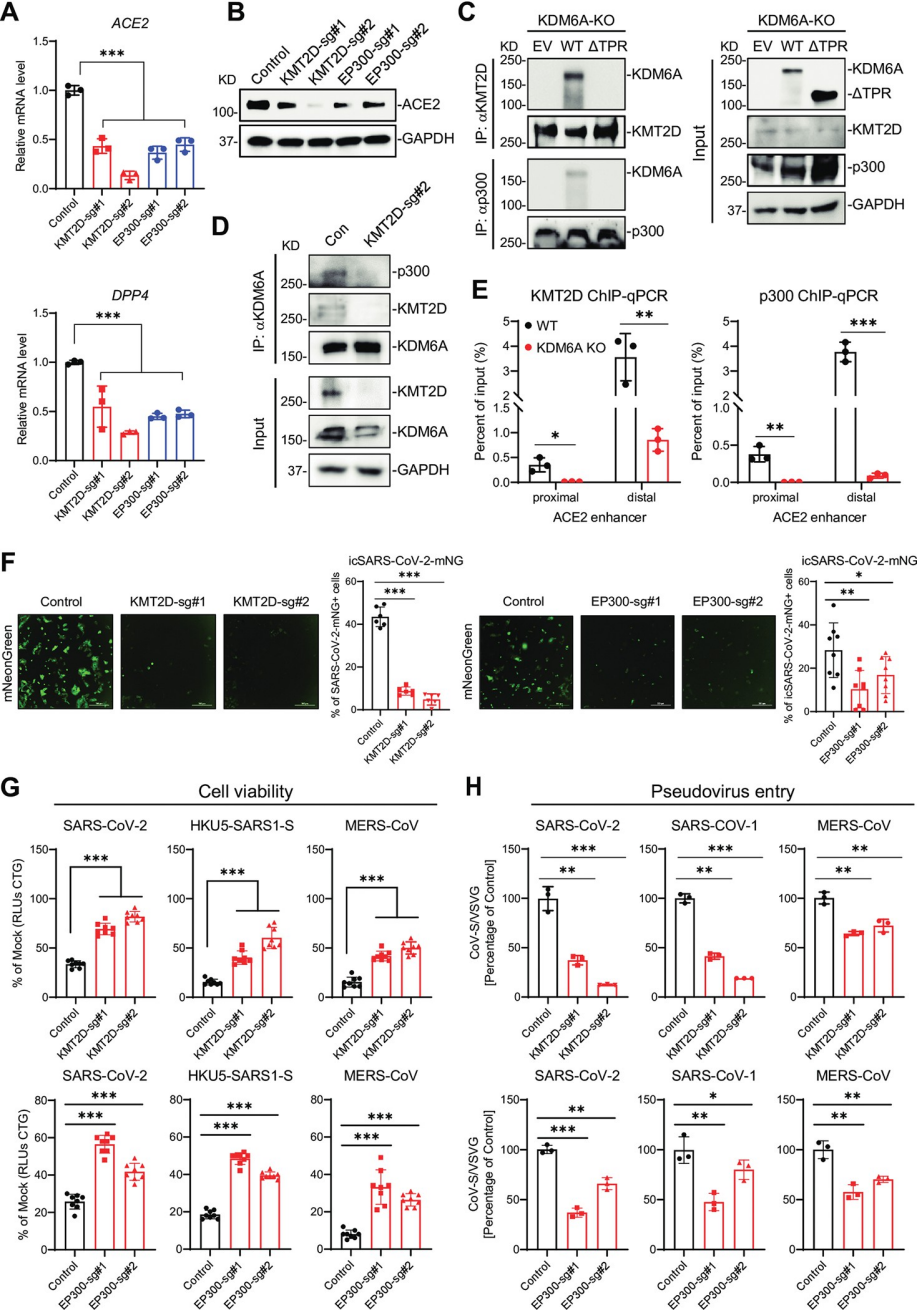
KDM6A recruits KMT2D and p300 through the TPR domain for receptor expression and viral entry. **(A-B)** ACE2 and DPP4 expression in KMT2D and EP300 polyclonal KO Huh7.5 cells at mRNA level (A) and protein level (B). **(C)** Co-immunoprecipitation assays in KDM6A KO Vero cells reconstituted with WT KDM6A, ΔTPR or empty vector (EV). **(D)** Co-immunoprecipitation assays in KMT2D polyclonal KO Huh7.5 cells. **(E)** ChIP-qPCR assays were performed using KMT2D and p300 antibody in WT and KDM6A KO Vero E6 cells to detect the binding sites in ACE2 proximal and distal enhancer regions. **(F)** KMT2D and EP300 polyclonal KO cells were infected with icSARS-CoV-2-mNG at an MOI of 1. Infected cells were imaged via fluorescence microscopy (left) and mNeonGreen expressing cell frequency was measured 2 dpi (right). Scale bar: 300 μm. **(G)** KMT2D and EP300 polyclonal Huh7.5 cells were infected with SARS-CoV-2 (left), HKU5-SARS-CoV-S (middle) and MERS-CoV (right) at an MOI of 0.2. Cell viability relative to a mock infected control was measured 3 dpi with CellTiter Glo. **(H)** KMT2D and EP300 polyclonal cells were infected with VSV pseudovirus (VSVpp): VSV-G, SARS-CoV-2-S (left), SARS-CoV-S (middle), and MERS-CoV-S (right). Luciferase relative to the VSVpp-VSV-G control was measured 1 dpi. Data were analyzed by one-way ANOVA with Tukey’s multiple comparison test. Shown are mean ± SEM. *p < 0.05, **p < 0.01, ***p < 0.001.

We next asked how KDM6A could regulate receptor expression in an enzyme-independent manner. The rescue experiments indicated that the TPR domain is essential for viral receptor expression (**Fig 2F–2H**). This domain is known to mediate the interaction between KDM6A and KMT2C or KMT2D (also called MLL3 and MLL4, respectively) [32,48]. In addition to KDM6A, CRISPR screens by us and others identified KMT2D and/or EP300 as critical pan-coronavirus pro-viral genes (**S3A and S3B Fig)** [27,29,30]. We next sought to define the potential role for KMT2D and EP300 in viral infection and receptor expression. To do this, we generated KMT2D and EP300 polyclonal KO Huh7.5 and Vero E6 cells using CRISPR/Cas9. Disruption of KMT2D and EP300 inhibited ACE2 and DPP4 expression in Huh7.5 cells (**Fig 3A and 3B**). We next sought to determine the mechanism by which ACE2 and DPP4 expression is regulated by the KDM6A/KMT2D/p300 complex. We performed immunoprecipitation assays in KDM6A KO cells reconstituted with WT KDM6A, ΔTPR mutant, or empty vector (EV). While ΔTPR was expressed at higher levels compared to WT KDM6A, immunoprecipitation of KMT2D or p300 captured only WT KDM6A but not ΔTPR (**Fig 3C**) This suggests that the TPR domain of KDM6A is required for association of KDM6A with KMT2D/p300. Further, KMT2D deficiency impaired the association between KDM6A and p300 (**Fig 3D**). We recently identified three putative *ACE2* enhancer sites, two distal and one proximal, that are regulated by BAF chromatin remodeling complex [49]. However, the enhancers of *DPP4* are undefined. To profile the occupancy of KMT2D and p300 at *ACE2* enhancer regions, we performed ChIP-qPCR for KMT2D and p300 in KDM6A KO Vero E6 cells. Genetic inactivation of KDM6A blocked the occupancy of KMT2D and p300 at the proximal and distal enhancers of *ACE2* (**Fig 3E**).

We next directly interrogated the role of KMT2D and EP300 in viral infection. Genetic depletion of either KMT2D or EP300 decreased icSARS-CoV-2-mNG infection in Huh7.5 and Vero E6 cells as measured by mNeonGreen (**Figs 3F and S3C**). Consistently, KMT2D or EP300 KO cells are resistant SARS-CoV-2, HKU5-SARS-CoV-S and MERS-CoV induced cell death (**Figs 3G and S3D**). Further, disruption of KMT2D and EP300 inhibited SARS-CoV, SARS-CoV-2, and MERS-CoV pseudovirus entry (**Figs 3H and S3E**). Collectively, these data demonstrate that KDM6A recruits KMT2D and p300 to form a complex that directly binds and regulates *ACE2* enhancers and thus expression.

### KDM6A-KMT2D-p300 axis is essential for murine coronavirus infection

**Fig 4:**
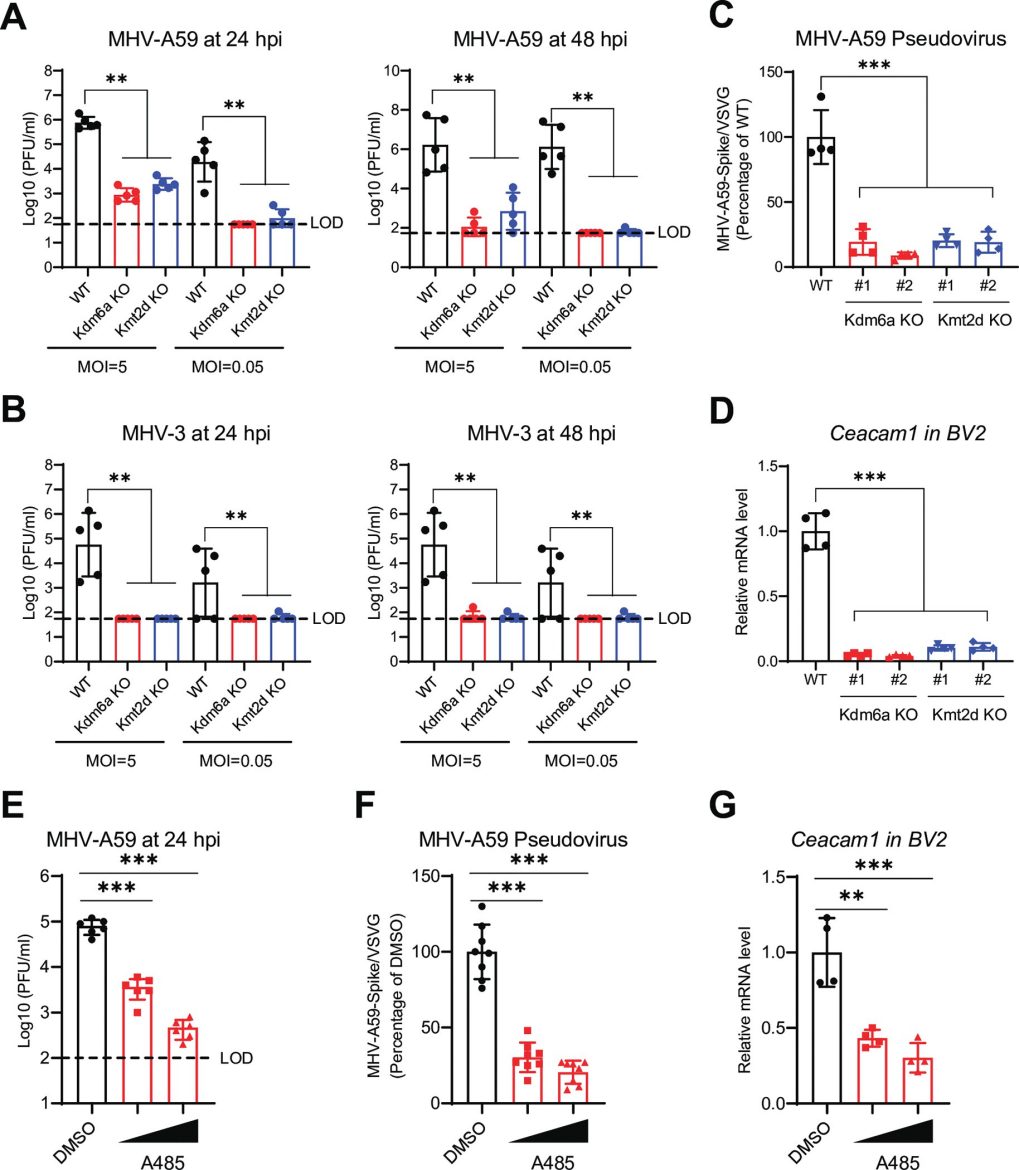
The KDM6A-KMT2D-p300 axis is essential for murine coronavirus infection. **(A-B)** Kdm6a and Kmt2d KO BV2 cells were infected with mouse hepatitis virus strain A59 (MHV-A59) (A) and MHV-3 (B) at 0.05 and 5 MOI for 24 hpi and 48 hpi. **(C)** Kdm6a and Kmt2d KO BV2 cells were infected with VSV peudovirus (VSVpp): VSV-G and MHV-A59-S. Luciferase relative to the VSVpp-VSV-G control was measured 1 dpi. **(D)** Ceacam1 expression in Kdm6a and Kmt2d KO BV2 cells. **(E)** BV2 cells were pretreated with A485 at 1 μM and 5 mM for 2 days and then infected with MHV-A59 at a MOI of 0.05. Virus production was measured by plaque assays. **(F)** BV2 cells were pretreated with A485 for 2 days and then infected with VSV peudovirus (VSVpp): VSV-G and MHV-A59-S. Luciferase relative to the VSVpp-VSV-G control was measured 1 dpi. **(G)** Ceacam1 expression in A485 treated BV2 cells. Dash line, limit of detection. Data were analyzed by one-way ANOVA with Tukey’s multiple comparison test. Shown are mean ± SEM. *p < 0.05, **p < 0.01, ***p < 0.001.

We next asked whether KDM6A/KMT2D/p300 axis is required for infection of other coronaviruses. To do this, we generated two independent single-cell KO clones for both *Kdm6a* and *Kmt2d* in BV2 microglia cells using CRISPR/Cas9 (**S4A and S4B Fig**). Genetic inactivation of Kdm6a or Kmt2d blocked mouse hepatitis virus A59 (MHV-A59) and MHV-3 infection in BV2 cells as measured by plaque assay (**Fig 4A and 4B**). Inactivation of Kdm6a and Kmt2d blocked MHV-A59 pseudovirus entry (**Fig 4C**). Given that KDM6A/KMT2D/p300 axis regulates receptor expression for highly pathogenic coronavirus, we hypothesized that Kdm6a/Kmt2d/Ep300 mediates MHV-A59 and MHV-3 receptor expression. Inactivation of Kdm6a or Kmt2d blocked expression of the MHV-A59 and MHV-3 receptor Ceacam1 as measured by qPCR (**Fig 4D**). Finally, we evaluated the effect of a potent and selective histone acetyltransferase activity inhibitor of p300, A485, on MHV infection [50]. Inhibition of p300 catalytic activity by A485 dose-dependently inhibited MHV-A59 replication and viral entry as measured by plaque assay and pseudovirus assay (**Fig 4E and 4F**). Consistently, A485 treatment dose-dependently inhibited Ceacam1 expression in BV2 cells (**Fig 4G**).

### p300 inhibition blocks ACE2 and DPP4 expression and viral infection in primary human airway cells

**Fig 5:**
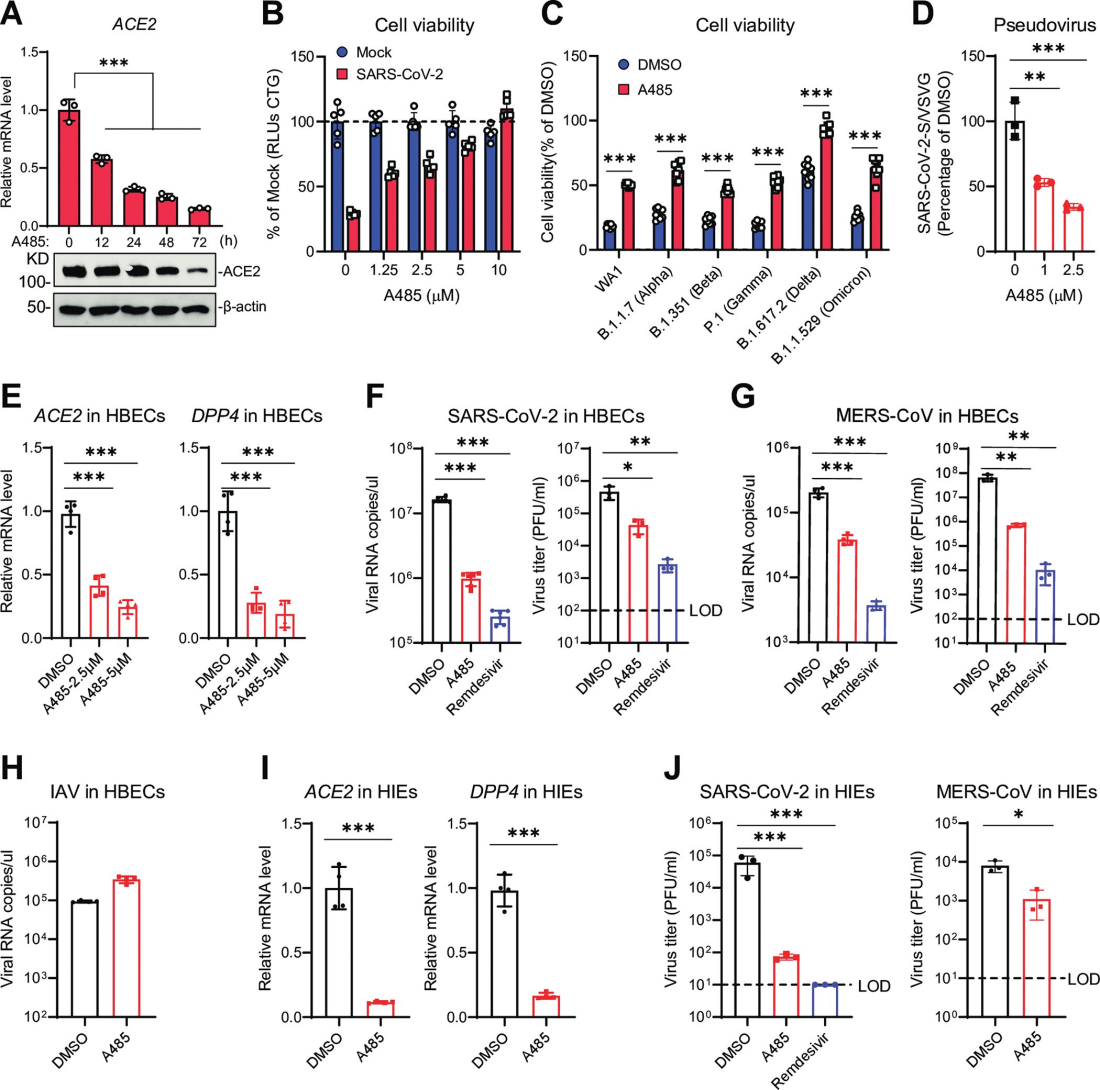
Small molecule inhibition of p300 downregulates ACE2 and DPP4 expression and blocks viral infection. **(A-B)** Vero E6 cells were treated with 1.25 μM GSK-J1 (A) or A485 (B) for the indicated times, ACE2 mRNA and protein levels were measured by RT-qPCR and immunoblot, respectively. **(C-D)** Vero E6 cells were pretreated with GSK-J1 (C) or A485 (D) inhibitors for 2 days and then infected with SARS-CoV-2 at an MOI of 0.2. Cell viability was measured at 3 dpi. **(E)** Vero E6 cells were pretreated with Comp12 for 2 days and then infected with the indicated SARS-CoV-2 variants at an MOI of 0.2. Cell viability was measured at 3 dpi. **(F)** Primary human bronchial epithelial cells (HBECs) were treated with A485 for 3 days and then measured the ACE2 and DPP4 expression by qPCR. **(G-H)** HBECs were pretreated A485 for 3 days then infected with SARS-CoV-2 and MERS at an MOI of 0.5 for 2 dpi. Virus replication was measured by qPCR (G) and plaque assays (H). Dash line, limit of detection. **(I)** Influenza virus replication at 1 dpi by qPCR in HBECs pretreated with A485 for 3 days. **(J)** Human intestinal enteroids (HIEs) were treated with 2.5 μM A485 for 3 days then measured the ACE2 and DPP4 expression by qPCR. **(K)** HIEs were pretreated A485 for 3 days then infected with SARS-CoV-2 and MERS at 10^5^ PFU/ml for 2 dpi. Virus replication was measured and plaque assays. Dash line, limit of detection. Data were analyzed by one-way ANOVA with Tukey’s multiple comparison test. Shown are mean ± SEM. *p < 0.05, **p < 0.01, ***p < 0.001.

To investigate the potential of KDM6A-KMT2D-p300 perturbation with human coronaviruses, we next evaluated the effect of the p300 catalytic inhibitor A485 on SARS-CoV-2 infection [50]. Notably, *ACE2* mRNA levels were reduced as early as 12 hours post treatment with A485, while maximal reduction of protein levels required 48–72 hours of treatment (**Fig 5A**). Consistent with genetic results, KDM6A demethylase activity inhibitor GSK-J1 had no effect on *ACE2* expression, suggesting KDM6A pro-viral activity is independent of demethylase activity (**Figs 2F and S5A**). Importantly, we observed dose-dependent inhibition of SARS-CoV-2-induced cell death with A485, whereas cell death was unaffected in GSK-J1 treated cells (**Figs 5B and S5B**). We next asked whether p300 inhibition could inhibit diverse SARS-CoV-2 variants. Similar to the anti-viral effects observed with the prototypic SARS-CoV-2 WA/01, A485 protected cells from infection against SARS-CoV-2 variants including B.1.1.7 (alpha), B.1.351 (beta), P.1 (gamma), B.1.617.2 (delta), and B.1.1.529 (Omicron) (**Fig 5C**). In addition, p300 inhibition with A485 reduced SARS-CoV-2 pseudovirus entry in a dose-dependent manner, whereas pseudovirus entry was unaffected by GSK-J1 (**Figs 5D and S5C**).

Next, we sought to evaluate the anti-viral effects of p300 catalytic inhibition in physiologically relevant primary cells. We first evaluated the impact of p300 inhibition in primary human bronchial epithelial cells (HBECs) cultured at air-liquid interface. Consistent with our findings in cell lines, p300 inhibition using A485 downregulated *ACE2* and *DPP4* expression in HBECs (**Fig 5E**). A485 treatment inhibited SARS-CoV-2 and MERS-CoV viral replication and virus production in HBECs as measured by qPCR and plaque assay (**Fig 5F and 5G**). However, influenza A virus (IAV A/WSN/1933) infection, which uses sialic acid as a receptor, was not reduced in A485-treated HBECs (**Fig 5H**). This suggests a degree of viral specificity of p300 catalytic inhibition in primary human airway cells.

Finally, we evaluated the p300 inhibitor efficacy in primary human intestinal enteroids (HIEs), which endogenously express ACE2 and DPP4. We pretreated HIEs with A485 inhibitor for 3 days in 3D culture, and then infected the cells with SARS-CoV-2 and MERS-CoV. Consistent with HBECs, A485 treatment inhibited ACE2 and DPP4 expression and viral infection (**Fig 5I and 5J**). Collectively, these data suggest that the KDM6A-KMT2D-p300 axis specifically regulates ACE2 and DPP4 expression and virus infection in primary human cells.

## Discussion

In this study, we demonstrate that KDM6A promotes diverse coronavirus infection by epigenetic regulation of three different coronavirus receptors (ACE2, DPP4 and Ceacam1) in a demethylase-independent manner. KDM6A recruits KMT2D and p300 through its TPR domain to regulate receptor expression and viral entry. Further, genetic inactivation of KDM6A impairs the occupancy of KMT2D and p300 at the ACE2 proximal and distal enhancers. KDM6A-KMT2D-p300 axis is conserved across mouse cell lines, a monkey cell line, two human cell lines, and two human primary cell types including airway epithelial cells. Inhibition of KDM6A/KMT2D/p300 complex activity, by either genetic or small molecule means, potently blocked ACE2, DPP4 and Ceacam1 expression and highly pathogenic coronavirus infection in cell line and primary human airway epithelial cells underscoring the potential prophylactic activity of KDM6A/KMT2D/p300 complex inhibitors for current and emerging coronavirus.

Recently, we and others have identified host factors that regulate ACE2 expression, including HMGB1, SMAD4, GATA6, DYRK1A, FXR, HNF1A/B, EP300, KDM6A and BAF complex [26,29,30,49,51,52]. Specifically, HMGB1 and BAF complexes regulate ACE2 chromatin accessibility and expression [26]. DYRK1A mediates ACE2 and DPP4 expression by promoting ACE2 promoter and enhancers accessibility seemingly independent of BAF and/or KDM6A-KMT2D-p300 complexes [49,51]. Here, we demonstrate that KDM6A complexes with KMT2D and p300 to regulate ACE2, DPP4 and Ceacam1 expression. Notably, KDM6A is not a sequence-specific DNA binding protein raising the question of how it targets receptor gene loci. Recent studies have demonstrated that the transcription factors HNF1A/B binds and recruits BAF complex to activate target gene transcription [49,53]. A recent study has identified that BAF complexes colocalize with KMT2D and lineage-determining transcription factors (LDTFs) resulting in enhancer activation [54]. In addition, KDM6A interacts with SMARCA4 in myeloid malignancies and loss of KDM6A reduces chromatin accessibility and enhancer activation [35]. We therefore speculate that KDM6A-KMT2D-p300 complexes bind and recruit chromatin remodeling complexes (i.e. BAF) and/or transcription factors (e.g. HNF1A/B) to the enhancers of *ACE2*, *DPP4* and *Ceacam*1 and thus promote diverse coronaviruses infection. While SMARCA4 regulates ACE2 expression in human cells, it does not regulate ACE2 expression in mice highlighting species-specific differences in gene regulation [49,53]. This limits the use of mice for studies of ACE2 epigenetic regulation. It is unknown whether the KDM6A-KMT2D-p300 axis mediates ACE2 or DPP4 expression in mice. Furthermore, additional work will be needed to delineate the interplay between the KDM6A-KMT2D-p300 complex and BAF or HNF1A/B in viral receptor expression.

Genes encoding KDM6A, KMT2D and EP300 are highly mutated in human cancers [55–59]. Our data show that reciprocal interactions between KDM6A, KMT2D and p300 are important for their recruitment to *ACE2* enhancers. In addition, inactivation of the catalytic activities of KMT2D and p300 abolished their ability to regulate enhancer activity in different cancers [57,59]. These findings support the possibility that KDM6A, KMT2D and p300 may regulate coronavirus infection and tumorigenesis by modulating enhancer activity. Beyond their roles in cancer and viral receptor expression, mutations in KDM6A and KMT2D have been identified in Kabuki syndrome, which is characterized by developmental delay [60,61]. The further study of how disease-associated mutations influence the recruitment and enhancer activation of KDM6A, KMT2D and p300 may predict the risk of coronavirus infection and disease severity.

Novel therapeutics are needed for current and emerging coronaviruses. Such therapeutics include direct-acting antivirals such as remdesivir, molnupiravir, nirmatrelvir/ritonavir, and spike monoclonal antibodies and host-directed therapeutics. While KDM6A, KMT2D, and p300 are essential for embryonic development, targeted deletion is well tolerated [62–64]. Notably, a novel CBP/p300 inhibitor is in a Phase I trial for metastatic prostate cancer (NCT04575766) [65]. Targeting the KDM6A/KMT2D/p300 axis offers several potential advantages in the regulation of receptor expression and diverse virus infection. First, targeting KDM6A-KMT2D-p300 offers protection from naturally occurring viral mutants, to which viral-targeted therapeutics and vaccines are more susceptible. Second, given the mechanism of KDM6A/KMT2D/p300 pathway in regulating gene expression in the host cell, viral antagonism is anticipated to be synergistic with existing, direct-acting antiviral and host immunomodulatory drugs. Third, by down regulating expression of multiple receptor genes, p300 inhibitor can inhibit diverse coronaviruses, including those that use ACE2 (HCoV-NL63, SARS-CoV, SARS-CoV-2 variants of concern and many bat sarbecoviruses), DPP4 (MERS-CoV and HKU4), and Ceacam1 (MHV-A59 and MHV-3). However, while targeting coronavirus receptor expression represents a potential anti-viral approach, the safety and tolerability of perturbing KDM6A-KMT2D-p300 and subsequently ACE2 and DPP4 in humans remains unclear and warrants future investigation [66]. Recent studies using FXR and BAF inhibitors highlight the potential of blocking ACE2 expression as an anti-viral approach [49,52]. However, given the pleiotropic functions of KDM6A-KMT2D-p300, ACE2, and DPP4, evaluation of the tolerability and efficacy of small molecules targeting KDM6A/KMT2D/p300 complex activity in suitable animal models and humans represent an important future direction. Taken together, our data suggest that targeting KDM6A-KMT2D-p300 axis is a promising strategy for development of broad-spectrum anti-viral therapeutics for diverse coronaviruses.

## Materials and methods

### Cells

HEK293T, Vero E6, Huh7.5 and L2 cells were cultured in Dulbecco’s Modified Eagle Medium (DMEM) with 10% heat-inactivated fetal bovine serum (FBS), and 1% Penicillin/Streptomycin unless otherwise indicated. Calu-3 cells were cultured in RPMI 1640 medium with Glutamax, 10% FBS, 1% Penicillin/Streptomycin and 16 ng/ml of hepatocyte growth factor (HGF, Stem Cell Technologies) to preserve viability and support robust growth. For Vero-E6 and Huh7.5 cells, 5 μg/ml of puromycin (Gibco) and 1500 μg/ml G418 (Gibco), were added as appropriate. For Calu-3 cells, 1 μg/ml of puromycin was added as appropriate.

### Air–liquid interface culture of HBECs and infection

Primary human bronchial epithelial cells (HBECs) were differentiated in air-liquid interface (ALI) culture as previously described [67]. HBECs were cultured in suspension in PneumaCult-Ex Plus Medium according to manufacturer’s instructions (STEMCELL Technologies). To generate ALI cultures, HBECs were plated on collagen-coated transwell inserts with a 0.4-micron pore size (Corning) at 5 × 10^4^ cells/ml per filter and inserted into 24-well culture plates. Cells were maintained for the first 3 days in PneumaCult-Ex Plus Medium, then changed to PneumaCult-ALI Medium (STEMCELL Technologies) containing the ROCK inhibitor Y-27632 for 4 days. Fresh medium, 100 μl in the apical chamber and 500 μl in the basal chamber, was provided every day. On day 7, medium at the apical chambers were removed, while basal chambers were maintained with 500 μl of PneumaCult-ALI Medium. Medium in the basal chamber was changed every 2 to 3 days. HBECs were maintained at ALI culture for 28 days, allowing them to differentiate.

Differentiated HBECs were pretreated with inhibitors or DMSO for 3 days at both apical and basal sides. HBECs were washed five times with PBS and inoculated with SARS-CoV-2, MERS-CoV and IAV from the apical side at an MOI of 0.5; the cell number per filter support was approximately 5x10^5^. After 1 h of incubation at 37°C, HBECs were rinsed with PBS twice to remove unbound viral particles. Infected HBECs were further maintained under ALI conditions at 37°C in a 5% CO2. At different time points, 100 μl of fresh medium were added to the apical surface and the cultures were incubated for 30 min at 37°C. The supernatants were collected at different times post virus-infection and the viruses were titrated by plaque assays on Vero-E6 cells. The cell lysates were harvested in Trizol for qPCR analysis.

### Human intestinal enteroid (HIE) culture and infection

Human intestinal enteroid (J2) derived from a biopsy specimen was kindly provided by Dr. Mary Estes from Baylor College of Medicine through the Texas Medical Center Digestive Diseases Center Enteroid Core. Protocols for culture, maintenance, and differentiation of HIEs were based on previous studies [68,69]. Briefly, frozen vials of HIEs were thawed out and resuspended in Cultrex Reduced Growth Factor Basement Membrane Extract (BME, equivalent of Matrigel;), Type 2, Select (R&D System). The BME mixture (25 μl/well) was plated as droplets into 24-well tissue culture plates and polymerized at 37°C for 10 min. 500 μl growth medium was added to each well and were changed every other day. After ~7–10 days, HIEs were expanded at 1 well:3 wells. For HIEs differentiation, growth medium was replaced with equal volume of differentiation media and incubated for 4–5 days with medium being changed every other day until use.

Differentiated human intestinal enteroids cells were pretreated with the indicated small molecules or DMSO for 3 days in 3D organoid culture. Enteroids were infected with SARS-CoV-2 or MERS-CoV at 10^5^ PFU/ml. After 1 h of incubation at 37°C, the media were replaced with fresh media. The cells with media were frozen and thawed. The supernatants were collected, and the viruses were titrated by plaque assays on Vero-E6 cells.

### Expression constructs and lentiviral infection

All constructs were PCR-amplified from cDNA using Q5 High-Fidelity DNA Polymerase with GC buffer (NEB). pInducer20 KDM6A WT, MT2 and ΔTPR mutant were previously described [32]. pLV-EF1A-ACE2 was a gift from Dr. Akiko Iwasaki. PLX307-DPP4 was purchased from Addgene (Cat#158451). For lentiviral transduction, cells were transduced at 50% confluency and selected with puromycin (5 μg/ml) or G418 (1600 μg/ml) 48 hours later.

### Viral stocks

To generate viral stocks, Vero E6 or Vero E6-ACE2-TMPRSS2 cells were inoculated with HKU5- SARS-CoV -S (NR-48814), SARS-CoV-2 isolate USA-WA1/2020 (NR-52281), B.1.1.298 (NR-53953), B.1.1.7 (NR-54000), B.1.351 (NR-54008), P.1 (NR-54982), B.1.617.2 (NR-55611), MERS-CoV (NR-48811) from BEI resources, B.1.1.529 was isolated from a patient at Yale New Haven Hospital [70] at an MOI of approximately 0.01 for three days to generate a P1 stock. The P1 stock was then used to inoculate Vero E6 or Vero E6-ACE2-TMPRSS2 cells for 1–3 days at approximately 50% cytopathic effects. Supernatant was harvested and clarified by centrifugation (450 *g* x 5 min) and filtered through a 0.45-micron filter, and then aliquoted for storage at -80°C. Virus titer was determined by plaque assay using Vero-E6 cells. To generate icSARS-CoV-2-mNG stocks, lyophilized icSARS-CoV-2-mNG was resuspended in 0.5 ml of deionized water and then 50 μl of virus was diluted in 5 ml media [47]. icSARS-CoV-2-mNG was provided by the World Reference Center for Emerging Viruses and Arboviruses (Galveston, TX). This was then added to 10^7^ Vero-E6 cells in a T175 flask. At 3 dpi, the supernatant was collected and clarified by centrifugation (450 *g* x 5 min), filtered through a 0.45-micron filter, and aliquoted for storage at -80°C. MHV-A59 (NR-4300) was from BEI resources and MHV-3 were obtained from ATCC, and propagate in L2 cells. All work with infectious virus was performed in a Biosafety Level 2 (BSL2) and BSL3 laboratory and approved by the Yale University Biosafety Committee.

### SARS-CoV-2, HKU5-SARS-CoV-S and MERS-CoV plaque assay

Vero E6 or Vero E6-ACE2-TMPRSS2 cells were seeded at 4 x 10^5^ cells/well in 12-well plates. The following day, the media was removed and replaced with 100 μl of 10-fold serial dilutions of virus. Plates were incubated at 37°C for 1 hour with gentle rocking. Subsequently, overlay media (DMEM, 2% FBS, 0.6% Avicel RC-581) was added to each well. At 2 dpi, plates were fixed with 10% formaldehyde for 30 min, stained with crystal violet solution (0.5% crystal violet in 20% ethanol) for 30 min, and then rinsed with deionized water to visualize plaques.

### SARS-CoV-2 TCID_50_ assay

Calu-3 cells were seeded at 2 × 10^5^ cells/well in 24-well plates. Two days later, cells were infected with SARS-CoV-2 at an MOI ~ 0.05. After 30 min incubation at 37°C, the unbound virus was removed, and cells were washed once with PBS. Fresh media was added, and cells were incubated for 24 hours. Plates were frozen and thawed to lyse cells. Viral lysates were serially diluted and added to eight wells in 96-well plates containing Vero E6 cells. Cytopathic effect (CPE) was determined visually at 3 dpi, and TCID_50_ per ml was calculated using the dilution factor required to produce CPE in half of the wells.

### Virus induced cell viability assay

Vero E6, Huh7.5 and Calu-3 cells were seeded at 1000 cells/well in 384 well plates. The following day, viruses were added to each well at a MOI 0.2–0.5. Plates were incubated at 37°C for 3 days (Vero-E6) or 4 days (Huh7.5 and Calu-3). Cell viability was measured by CellTiter Glo (Promega).

### Mouse hepatitis virus (MHV) plaque assay

L2 cells were seeded at 8 x 10^5^ cells/well in 6-well plates. The following day, the media was removed and replaced with 200 μl of 10-fold serial dilutions of virus. Plates were incubated at room temperature for 1 hour with gentle rocking. Subsequently, overlay media (DMEM, 2% FBS, 0.6% Avicel RC-581) was added to each well. At 2 dpi, plates were fixed and stained with 0.2% crystal violet in 70% ethanal for 30 min, and then rinsed with deionized water to visualize plaques.

### SARS-CoV-2 fluorescent reporter virus assay

Cells were plated at 2.5 x 10^3^ cells per well in a 384-well plate and then the following day, icSARS-CoV-2-mNG was added at an MOI of 1.0 [47]. Infected cell frequencies as measured by mNeonGreen expression were assessed at 2 dpi by high content imaging (Cytation 5, BioTek) configured with bright field and GFP cubes. Total cell numbers were quantified by Gen5 software of brightfield images. Object analysis was used to determine the number of mNeon Green positive cells. The percentage of infection was calculated as the ratio between the number of mNeonGreen+ cells and the total number of cells in brightfield.

### Generation of polyclonal KO cell lines

Double-stranded oligonucleotides corresponding to the target sequences were cloned into the lentiCRISPR-V2 vector and co-transfected packaging plasmids into 293T cells. Lentiviral particles were collected and used to transduce Vero E6, Huh7.5 or Calu-3 cells. The infected cells were selected with puromycin for 2 weeks before additional experiments were performed. The guide RNA target sequences are shown below:

KDM6A sgRNA#1: CCTAGCAATTCAGTAACACA
KDM6A sgRNA#2: TCTTTGTATGAACAGCTGGG
KMT2D sgRNA#1: CTGTGAATGGCAGAATAGTT
KMT2D sgRNA#2: CGCCGGCTTGTCTACCTCTG
EP300 sgRNA#1: AATATGCAGTACCCAAACCC
EP300 sgRNA#2: CACTGTCGCACAATGAAGAA

### Generation of KDM6A knockout and complemented cells

Vero E6 KDM6A KO cells were generated by lipofection of Cas9-RNPs. CRISPR guide RNA (gRNA) were synthesized by IDT. gRNAs were complexed at a 1:1 molar ratio with ATTO550 labelled tracrRNA in TE buffer by heating at 95°C for 5 min followed by cooling to room temperature to form crRNA: tracrRNA duplexes, Alt-R Cas9 enzyme was combined with crRNA: tracrRNA duplex at room temperature for 20 min to form ribonucleoprotein (RNP) complexes in Opti-MEM with 50 ul total volume. Complexes were mixed with Lipofectamine RNAiMAX for 10 min at room temperature before transfection was performed. Single cells were then sorted by flow cytometry and KDM6A knockout was confirmed by western blot.

KDM6A KO clones were complemented by lentiviral transduction of pInducer20 vector or containing full-length KDM6A, a catalytically dead KDM6A mutant (MT2), a lacking the TPR (ΔTPR) mutant with a C-terminal V5 tag. Two days post transduction, G418 was added, and cells were selected for seven days. The cells were treated with Doxycycline (100ng/ml) for 2 days before western blot for KDM6A expression of in complemented cells.

KDM6A KO clones were transduced with pLenti-hACE2-Puro (Addgene: #155295) and pLEX307-DPP4-Puro (Addgene: #158451) to overexpress human ACE2 and human DPP4, respectively. Two days post transduction, Puromycin was added, and cells were selected for 5 days. ACE2 and DPP4 expression was detected by western blot.

### Generation of Kdm6a and Kmt2d knockout BV2 cells

Kdm6a and Kmt2d BV2 knockout cells were generated at the Genome Engineering and iPSC center at Washington University School of Medicine. BV2 cells were nucleofected with Cas9 and a Kdm6a-specific sgRNA (5’-GAAACCTCAVGAACCCGAA-3’) or Kmt2d-specific sgRNA (5’-GAGGTCTCCGTCCCCGGTTC-3’). Cells were then single cell sorted and genomic DNA was extracted and amplified. Clones were screened for frameshifts by sequencing the target region with Illumina MiSeq at approximately 500x coverage. Two ΔKdm6a and two ΔKmt2d BV2 clones were selected for subsequent experiments (clone #1and clone #2).

The targeted Kdm6a and Kmt2d mutations in these clones are shown below:

WT CATGAAGATGGCGCCAGGATGAAGGCCCTGCTG
Kdm6a KO#1 (-2bp) CATGAAGATGGCGCCAGGA-—AAGGCCCTGCTG
Kdm6a KO#2 (-1bp) CATGAAGATGGCGCCAGG -TGAAGGCCCTGCTG
WT GAGGGTCTCCGTCCCCGGTTCTGGGGGTTCAGG
Kmt2d KO#1 (-7bp) GAGGGTCTCCGTCCC—-—-—-—GGGGGTTCAGG
Kmt2d KO#2 (-2bp) GAGGGTCTCCGTCCCCGG-—CTGGGGGTTCAGG

### KDM6A and p300 inhibitor treatment for cell lines

GSK-J1 was purchased from Sigma-Aldrich (SML0709-5MG); A485 was purchased from Cayman Chemical Company (Cat#24119). Vero E6 cells (1.5 x10^4^) were pretreated with the indicated concentration of GSK-J1 and A485 for 48 hours and then infected with SARS-CoV-2 or MERS-CoV at an MOI of 0.2. Cell viability was quantified by CellTiter Glo at 3 dpi. Vero E6, Huh7.5 and Calu-3 cells (1x10^6^) were pretreated with 2.5 μM GSK-J1 or A485 for 48 hours, then the ACE2 expression was detected by RT-qPCR and western blot. Cytotoxicity was not observed in these cell lines during the time and concentration of drug used.

### Pseudovirus production

VSV-based pseudotype viruses were produced as described previously [26,71]. Plasmids encoding codon-optimized form of SARS1-S glycoprotein, and MERS SΔCT glycoproteins lacking cytoplasmic tail were previously described [72], pCAGGS-MHV-A59-Spike was a gift from Dr. Thomas Gallagher (University of Wisconsin). Vector pCAGGS containing the SARS-Related Coronavirus 2, Wuhan-Hu-1 Spike Glycoprotein Gene, NR-52310, was produced under HHSN272201400008C and obtained through BEI Resources, NIAID, NIH. Briefly, 293T cells were transfected with pCAGGS or pcDNA3.1 vector expressing the CoV spike glycoprotein and then inoculated with a replication-deficient VSV virus that contains expression cassettes for Renilla luciferase instead of the VSV-G open reading frame. After an incubation period of 1 h at 37°C, the inoculum was removed, and cells were washed with PBS before media supplemented with anti-VSV-G clone I4 was added to neutralize residual input virus (no antibody was added to cells expressing VSV-G). Pseudotyped particles were harvested 24 hours post inoculation, clarified from cellular debris by centrifugation and stored at -80°C before use.

### Pseudovirus entry assay

1x10^4^ Vero E6, Huh7.5, Calu-3 or BV2 cells were seeded in 100 μl total volume each well of a black-walled clear bottom 96-well plate. The following day spike expressing VSV pseudovirus was added at 1:10 final concentration volume/volume and incubated for one day. Cells were lysed with Renilla Luciferase Assay System (Promega) according to manufacturer instructions. Luciferase activity was measured using a microplate reader (BioTek Synergy or Cytation 5).

### RT-qPCR

Total RNA was isolated from cells using Direct-zol RNA MiniPrep Plus kit and and 1 μg RNA used for cDNA synthesis. Quantitative PCR was carried out using specific primers and probes for SARS-CoV-2 nucleocapsid (N1) genes (Forward: 5′-GACCCCAAAATCAGCGAAAT-3′; Reverse: 5′-TCTGGTTACTGCCAGTTGAATCTG-3′; Probe: 5′-6FAM-ACCCCGCATTACGTTTGGTGGACC-BHQ1-3′)

IAV-PA (Forward: 5′-GGCCGACTACACTCTCGATGA-3′; Reverse: 5′-TGTCTTATGGTGAATAGCCTGGTTT-3′)

ACE2 (Forward: 5′-GGGATCAGAGATCGGAAGAAGA-3′; Reverse: 5′-AAGGAGGTCTGAACATCATCAGTG-3′)

DPP4 (Forward: 5′-GAATTATCCGGTCGAGTTTT-3′; Reverse: 5′-GCCATCCTTTTAAAGAAGAG-3′)

ACTIN (Forward:5′-GAGCACAGAGCCTCGCCTTT-3′; Reverse:5′-ATCATCATCCATGGTGAGCTGG-3′)

Mouse Actin (Forward: 5′- ACTGTCGAGTCGCGTCCA -3′; Reverse: 5′- ATCCATGGCGAACTGGTGG-3′)

Mouse Ceacam1 (Forward: 5′-CCTCTATTCCAGGAAGTCTGGC-3’; Reverse: 5′-GTTCAGGACAGTGTATGCGACG-3′)

### Coimmunoprecipitation

Cells were lysed in 1 ml Nonidet P-40 lysis buffer (20 mM Tris-HCl [pH 7.4], 150 mM NaCl, 1 mM EDTA, 1% Nonidet P-40, 10 mg/ml aprotinin, 10 mg/ml leupeptin, and 1 mM PMSF). For each immunoprecipitation, a 0.4-ml aliquot of lysate was incubated with 0.5–2 μg of the indicated antibody or control IgG and 25 μl of a 1:1 slurry of Protein-G Sepharose (Goldbio) for at least 3 h. The Sepharose beads were washed three times with 1 ml of lysis buffer containing 500 mM NaCl. The precipitates were fractionated on SDS-PAGE, and immunoblot analysis was performed following standard methods.

### Western blot

Cells were collected and lysed in Nonidet P-40 lysis buffer. The cell lysates or Co-IP precipitates were fractionated on SDS-PAGE and transferred to a PVDF membrane. Immunoblotting analyses were performed with the indicated antibodies and visualized with horseradish peroxidase-coupled goat anti-mouse/rabbit IgG using a chemiluminescence detection system (BioRad ChemiDoc MP).

### ChIP-qPCR

KMT2D and p300 ChIPs were performed using ChIP-IT High Sensitivity kit (Active Motif Cat#53040) following the manufacturer’s instructions. Briefly, Vero E6 cells (40 x 10^6^/per assay) were fixed initially with 25 mM DMA (Sigma-Aldrich Cat# 285625) at room temperature for 1 hour, and subsequently with 1% formaldehyde at room temperature for 10 min. Quench the formaldehyde by adding Glycine diluted to a final concentration of 125 mM at room temperature for 5 min. Fixed cells were washed twice with PBS and cell pellet were collected stored at -80°C before ChIP assays. The fixed cells were lysed with provided lysis solution supplemented with protease inhibitors. Next, chromatin was sonicated to obtain DNA fragments within the recommended 200–1200 bp range. In total, 100 μg of sheared chromatin was then incubated with 4 μg of antibody against KMT2D (Invitrogen Cat#PA5-115579), or p300 (Invitrogen Cat#33–7600 Clone NM-11) overnight at 4°C with rotation. Following incubation with Protein G agarose beads, bound chromatin was washed, eluted, and purified following the manufacturer’s protocols. ChIP DNAs were analyzed by qPCR. Data were normalized to percentage of input DNA. ACE2 enhancer qPCR primer as following: Proximal ACE2 enhancer: (Forward: 5’-ACCTAGGCTAGACCAGGCTGT-3’, Reverse: 5’-TCCATTCTGTTTCTCCATTTCCCA-3’); Distal ACE2 enhancer: (Forward: 5’-ATGACCCTGAACCCCAAACTC-3’, Reverse: 5’-GCTGGCCACAGTAAGGATGC-3’).

### RNA-seq

Total cellular RNA was extracted using Direct-zol RNA MiniPrep Kit and submitted to the Yale Center for Genome Analysis for library preparation. RNA-seq libraries were sequenced on an Illumina NovaSeq 6000 instrument with the goal of at least 25 × 10^6^ reads per sample. Reads were aligned to reference genome chlSab2, NCBI annotation release 100, using STAR aligner v2.7.3a with parameters–winAnchorMultimapNmax 200–outFilterMultimapNmax 100–quantMode GeneCounts. Differential expression was obtained using the R package DESeq2 v1.32 [73]. Bigwig files were generated using deeptools v3.1.3 [74] with parameter–normalizeUsing RPKM.

### Quantification and statistical analysis

All statistical analysis was performed in Prism GraphPad version 8 unless otherwise stated. Error bars represent the standard error of the mean (SEM). Data was analyzed using unpaired Student’s t-tests or one-way ANOVA with Tukey’s multiple comparison test. Viral shedding over time was analyzed by repeated-measures ANOVA. All statistically analyzed pairwise comparisons are indicated with a bar and the p-value is represented by a symbol. (* p-value <0.05, ** p-value <0.01, *** p-value <0.0001) The absence of a bar indicated no statistical pairwise comparisons were made.

## Supporting information

S1 Fig KDM6A is required for highly pathogenic coronavirus infection in Huh7.5 cells. **(A)** KDM6A expression in KDM6A polyclonal KO Huh7.5 cells. **(B)** KDM6A polyclonal KO Huh7.5 cells were infected with icSARS-CoV-2-mNeonGreen at an MOI of 1. Infected cells were imaged via fluorescence microscopy (left) and mNeonGreen expressing cell frequency was measured 2 dpi (right). Scale bar: 300 μm. **(C)** Huh7.5 cells were infected with SARS-CoV-2 at an MOI of 0.1 for 1 dpi. Virus titer was measured by plaque assays. **(D)** KDM6A polyclonal Huh7.5 E6 cells were infected with SARS-CoV-2 (left), HKU5-SARS-CoV-1-S (middle) and MERS-CoV (right) at an MOI of 0.2. Cell viability relative to a mock infected control was measured 3 dpi with CellTiter Glo. (E) KDM6A polyclonal Huh7.5 E6 cells were infected with VSV peudovirus (VSVpp): VSV-G, SARS-CoV-2-S (left), SARS-CoV-1-S (middle), and MERS-CoV-S (right). Luciferase relative to the VSVpp-VSV-G control was measured 1 dpi. Data were analyzed by one-way ANOVA with Tukey’s multiple comparison test. Shown are mean ± SEM. *p < 0.05, **p < 0.01, ***p < 0.001.
(TIF)

S2 Fig KDM6A regulates ACE2 and DPP4 in Vero E6 cells. **(A)** Volcano plot for RNA sequencing of control and KDM6A polyclonal KO Vero E6 cells. The x axis shows log2 fold change and the y axis shows -log10 of the adjusted P value (adj.P) as calculated by DESeq2. **(B)** Top gene sets, which significantly enriched in the differentially expression genes from KEGG. **(C)** ACE2 expression level in KDM6A KO cells rescued with human ACE2. **(D)** DPP4 expression level in KDM6A KO cells rescued with human DPP4. **(E)** VSVpp-MERS-S pseudovirus entry in WT Vero E6 cells and KDM6A KO cells rescued with human ACE2. **(F)** VSVpp-SARS-CoV-2-S pseudovirus entry in WT Vero E6 cells and KDM6A KO cells rescued with human DPP4. Luciferase relative to the VSVpp-VSV-G control was measured 1 dpi. Data were analyzed by one-way ANOVA with Tukey’s multiple comparison test. Shown are mean ± SEM. *p < 0.05, **p < 0.01, ***p < 0.001.
(TIF)

S3 Fig KMT2D and EP300 is required for highly pathogenic coronavirus infection in Vero E6 cells. **(A)** CRISPR screen z-score for highly pathogenic coronavirus infection in Vero E6 cells. **(B)** Gene rank for KDM6A, KMT2D and EP300 in different CRISPR screens. **(C)** KMT2D and EP300 polyclonal KO Vero E6 cells were infected with SARS-CoV-2-mNeonGreen at an MOI of 1. Infected cells were imaged via fluorescence microscopy (left) and mNeonGreen expressing cell frequency was measured 2 dpi (right). Scale bar: 300 μm. **(D)** Vero E6 cells were infected with SARS-CoV-2 (left), HKU5-SARS-CoV-1-S (middle) and MERS-CoV (right) at an MOI of 0.2. Cell viability relative to a mock infected control was measured 3 dpi with CellTiter Glo. **(E)** KMT2D and EP300 polyclonal KO Vero E6 cells were infected with VSV peudovirus (VSVpp): VSV-G, SARS-CoV-2-S (left), SARS-CoV-1-S (middle), and MERS-CoV-S (right). Luciferase relative to the VSVpp-VSV-G control was measured 1 dpi. Data were analyzed by one-way ANOVA with Tukey’s multiple comparison test. Shown are mean ± SEM. *p < 0.05, **p < 0.01, ***p < 0.001.
(TIF)

S4 Fig CRISPR-Cas9 mediated Kdm6a and Kmt2d KO BV2 cells. **(A)** Sequence alignment of two Kdm6a KO clones in BV2 cells with 2 bp and 1 bp nucleotide deletion, respectively. **(B)** Sequence alignment of two Kmt2d KO clones in BV2 cells with 7 bp and 2 bp nucleotide deletion, respectively.
(TIF)

S5 Fig GSK-J1 has no effects on SARS-CoV-2 infection. **(A)** Vero E6 cells were treated with 1.25 μM GSK-J1 for the indicated times, ACE2 mRNA and protein levels were measured by RT-qPCR and immunoblot, respectively. **(B)** Vero E6 cells were pretreated with GSK-J1 for 2 days and then infected with SARS-CoV-2 at an MOI of 0.2. Cell viability was measured at 3 dpi. **(C)** Vero E6 cells were pretreated with GSK-J1 for 2 days and then infected with VSV pseudovirus (VSVpp): VSV-G and SARS-CoV-2-S. Luciferase relative to the VSVpp-VSV-G control was measured 1 dpi. Data were analyzed by one-way ANOVA with Tukey’s multiple comparison test. Shown are mean ± SEM. *p < 0.05, **p < 0.01, ***p < 0.001.
(TIF)
