# Supplementary figures and images for: The KDM6A-KMT2D-p300 axis regulates susceptibility to diverse coronaviruses by mediating viral receptor expression
Source: PLoS Pathog. 2023 Jul 6;19(7):e1011351. doi: 10.1371/journal.ppat.1011351 (PMC10325096; doi:10.1371/journal.ppat.1011351)

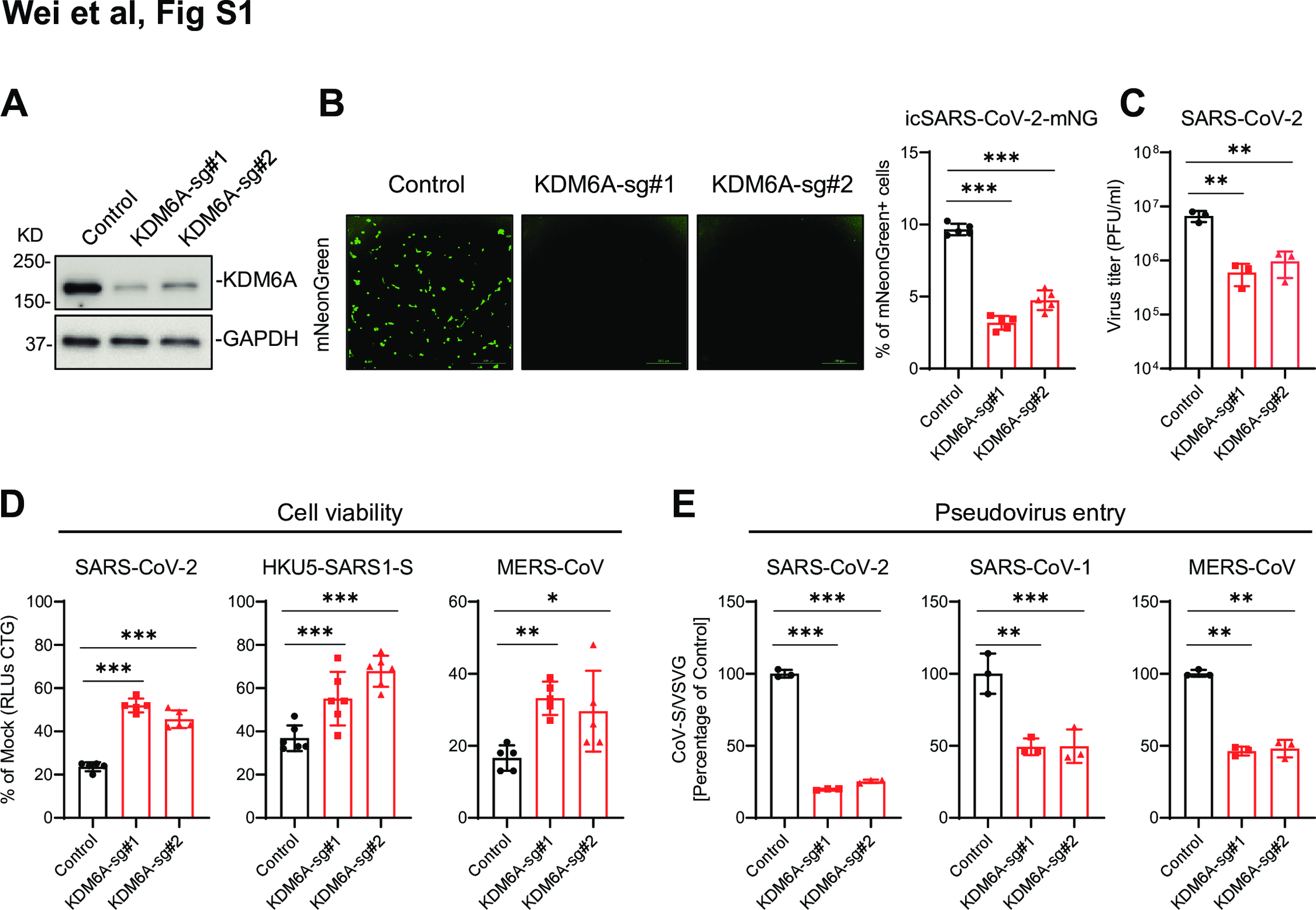

Supplement: S1 Fig — (A) KDM6A expression in KDM6A polyclonal KO Huh7.5 cells. (B) KDM6A polyclonal KO Huh7.5 cells were infected with icSARS-CoV-2-mNeonGreen at an MOI of 1. Infected cells were imaged via fluorescence microscopy (left) and mNeonGreen expressing cell frequency was measured 2 dpi (right). Scale bar: 300 μm. (C) Huh7.5 cells were infected with SARS-CoV-2 at an MOI of 0.1 for 1 dpi. Virus titer was measured by plaque assays. (D) KDM6A polyclonal Huh7.5 E6 cells were infected with SARS-CoV-2 (left), HKU5-SARS-CoV-1-S (middle) and MERS-CoV (right) at an MOI of 0.2. Cell viability relative to a mock infected control was measured 3 dpi with CellTiter Glo. (E) KDM6A polyclonal Huh7.5 E6 cells were infected with VSV peudovirus (VSVpp): VSV-G, SARS-CoV-2-S (left), SARS-CoV-1-S (middle), and MERS-CoV-S (right). Luciferase relative to the VSVpp-VSV-G control was measured 1 dpi. Data were analyzed by one-way ANOVA with Tukey’s multiple comparison test. Shown are mean ± SEM. *p < 0.05, **p < 0.01, ***p < 0.001. (TIF) [file ppat.1011351.s001.tif]

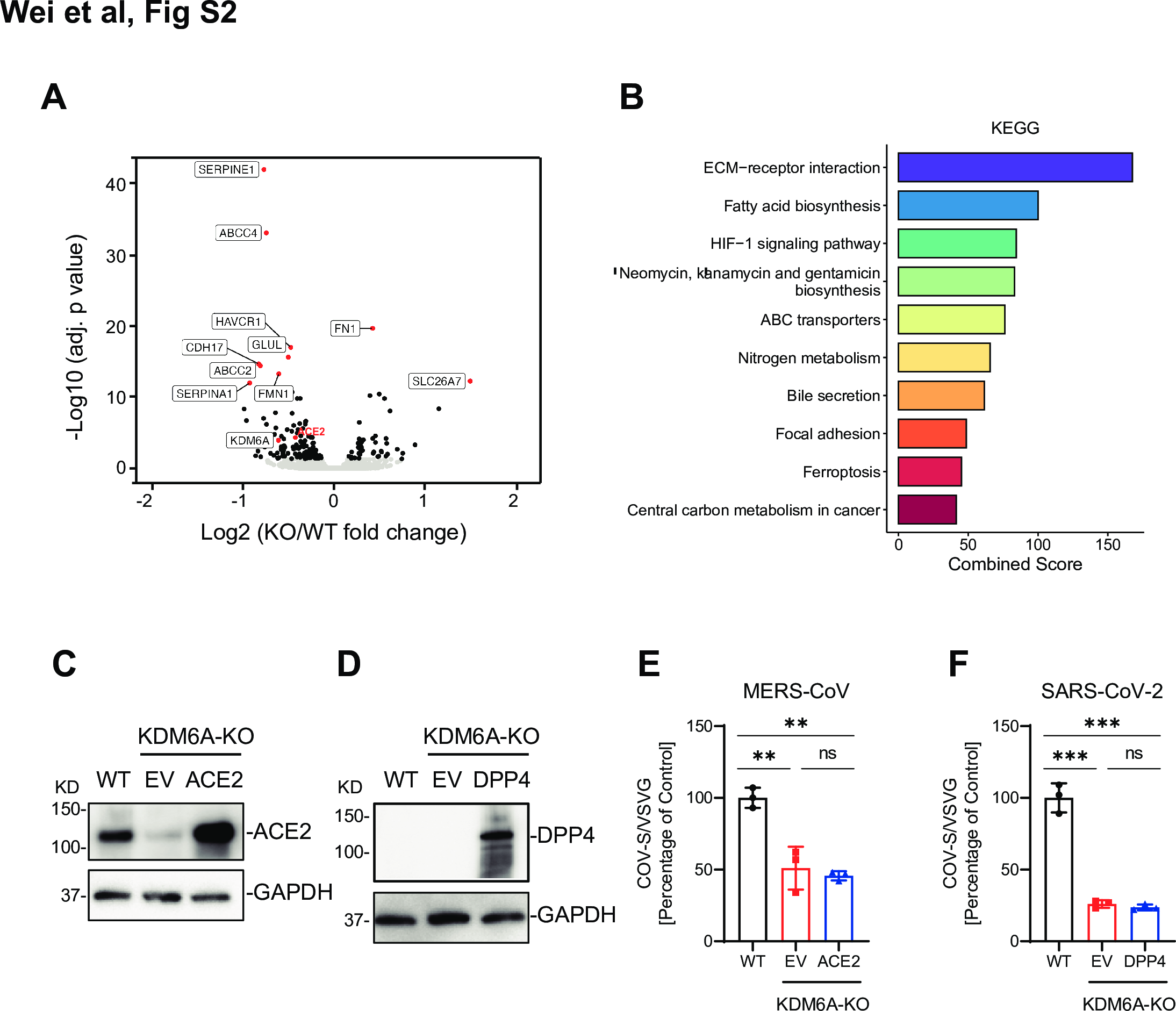

Supplement: S2 Fig — (A) Volcano plot for RNA sequencing of control and KDM6A polyclonal KO Vero E6 cells. The x axis shows log2 fold change and the y axis shows -log10 of the adjusted P value (adj.P) as calculated by DESeq2. (B) Top gene sets, which significantly enriched in the differentially expression genes from KEGG. (C) ACE2 expression level in KDM6A KO cells rescued with human ACE2. (D) DPP4 expression level in KDM6A KO cells rescued with human DPP4. (E) VSVpp-MERS-S pseudovirus entry in WT Vero E6 cells and KDM6A KO cells rescued with human ACE2. (F) VSVpp-SARS-CoV-2-S pseudovirus entry in WT Vero E6 cells and KDM6A KO cells rescued with human DPP4. Luciferase relative to the VSVpp-VSV-G control was measured 1 dpi. Data were analyzed by one-way ANOVA with Tukey’s multiple comparison test. Shown are mean ± SEM. *p < 0.05, **p < 0.01, ***p < 0.001. (TIF) [file ppat.1011351.s002.tif]

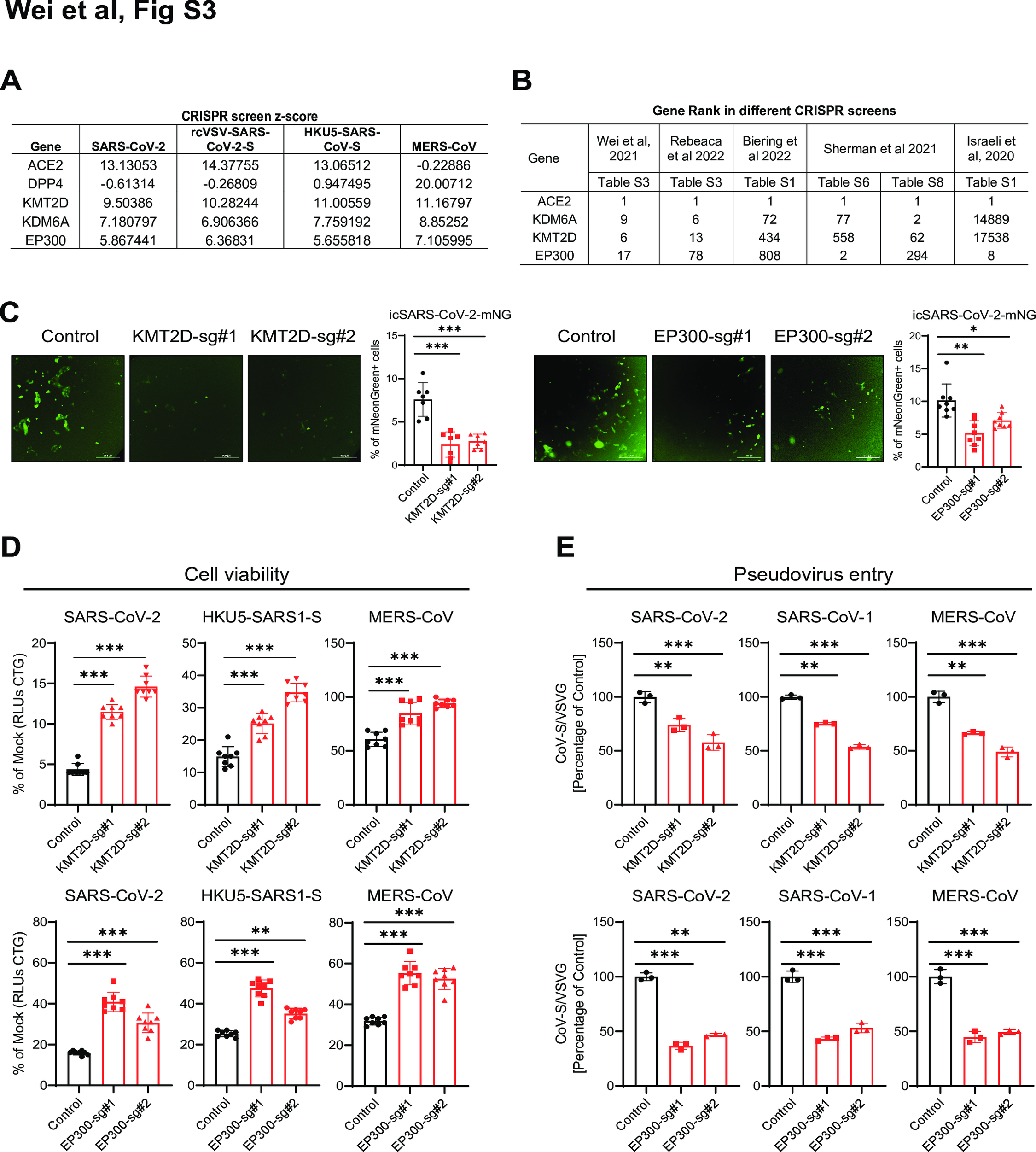

Supplement: S3 Fig — (A) CRISPR screen z-score for highly pathogenic coronavirus infection in Vero E6 cells. (B) Gene rank for KDM6A, KMT2D and EP300 in different CRISPR screens. (C) KMT2D and EP300 polyclonal KO Vero E6 cells were infected with SARS-CoV-2-mNeonGreen at an MOI of 1. Infected cells were imaged via fluorescence microscopy (left) and mNeonGreen expressing cell frequency was measured 2 dpi (right). Scale bar: 300 μm. (D) Vero E6 cells were infected with SARS-CoV-2 (left), HKU5-SARS-CoV-1-S (middle) and MERS-CoV (right) at an MOI of 0.2. Cell viability relative to a mock infected control was measured 3 dpi with CellTiter Glo. (E) KMT2D and EP300 polyclonal KO Vero E6 cells were infected with VSV peudovirus (VSVpp): VSV-G, SARS-CoV-2-S (left), SARS-CoV-1-S (middle), and MERS-CoV-S (right). Luciferase relative to the VSVpp-VSV-G control was measured 1 dpi. Data were analyzed by one-way ANOVA with Tukey’s multiple comparison test. Shown are mean ± SEM. *p < 0.05, **p < 0.01, ***p < 0.001. (TIF) [file ppat.1011351.s003.tif]

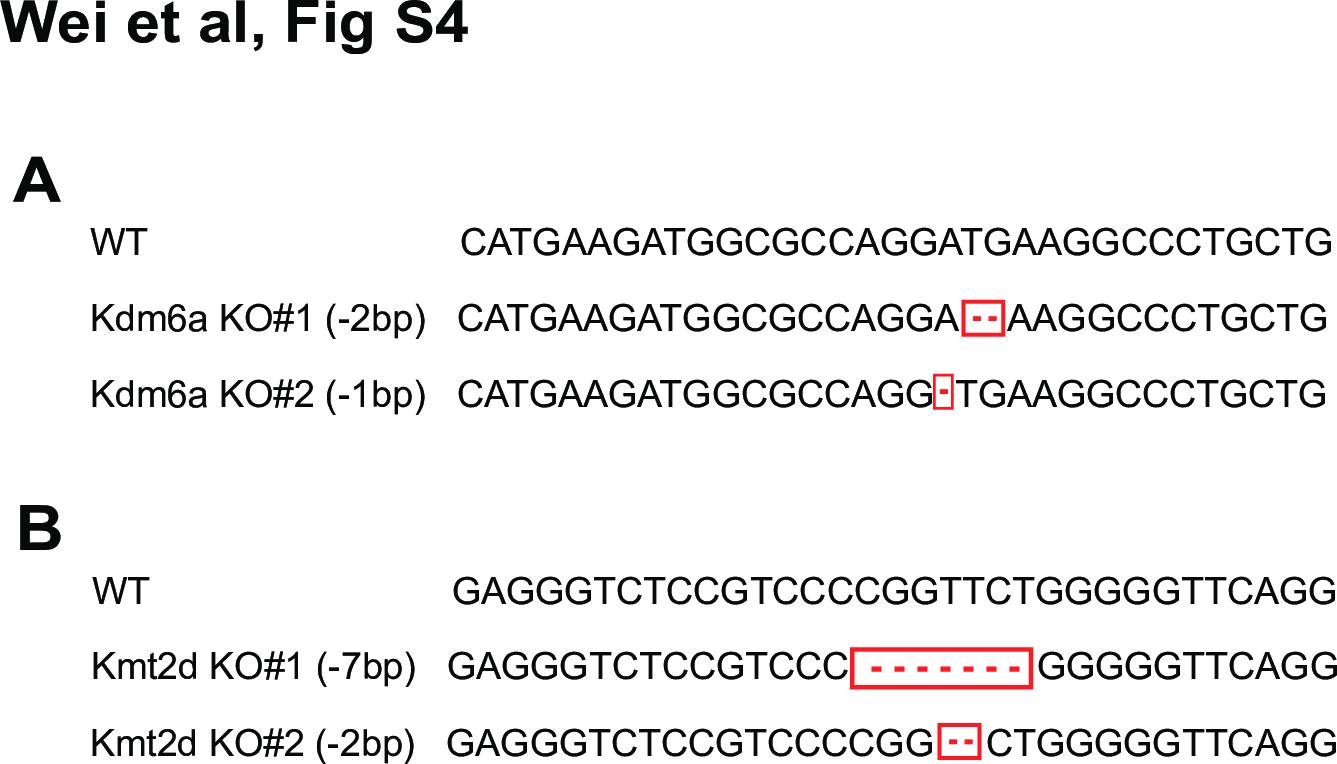

Supplement: S4 Fig — (A) Sequence alignment of two Kdm6a KO clones in BV2 cells with 2 bp and 1 bp nucleotide deletion, respectively. (B) Sequence alignment of two Kmt2d KO clones in BV2 cells with 7 bp and 2 bp nucleotide deletion, respectively. (TIF) [file ppat.1011351.s004.tif]

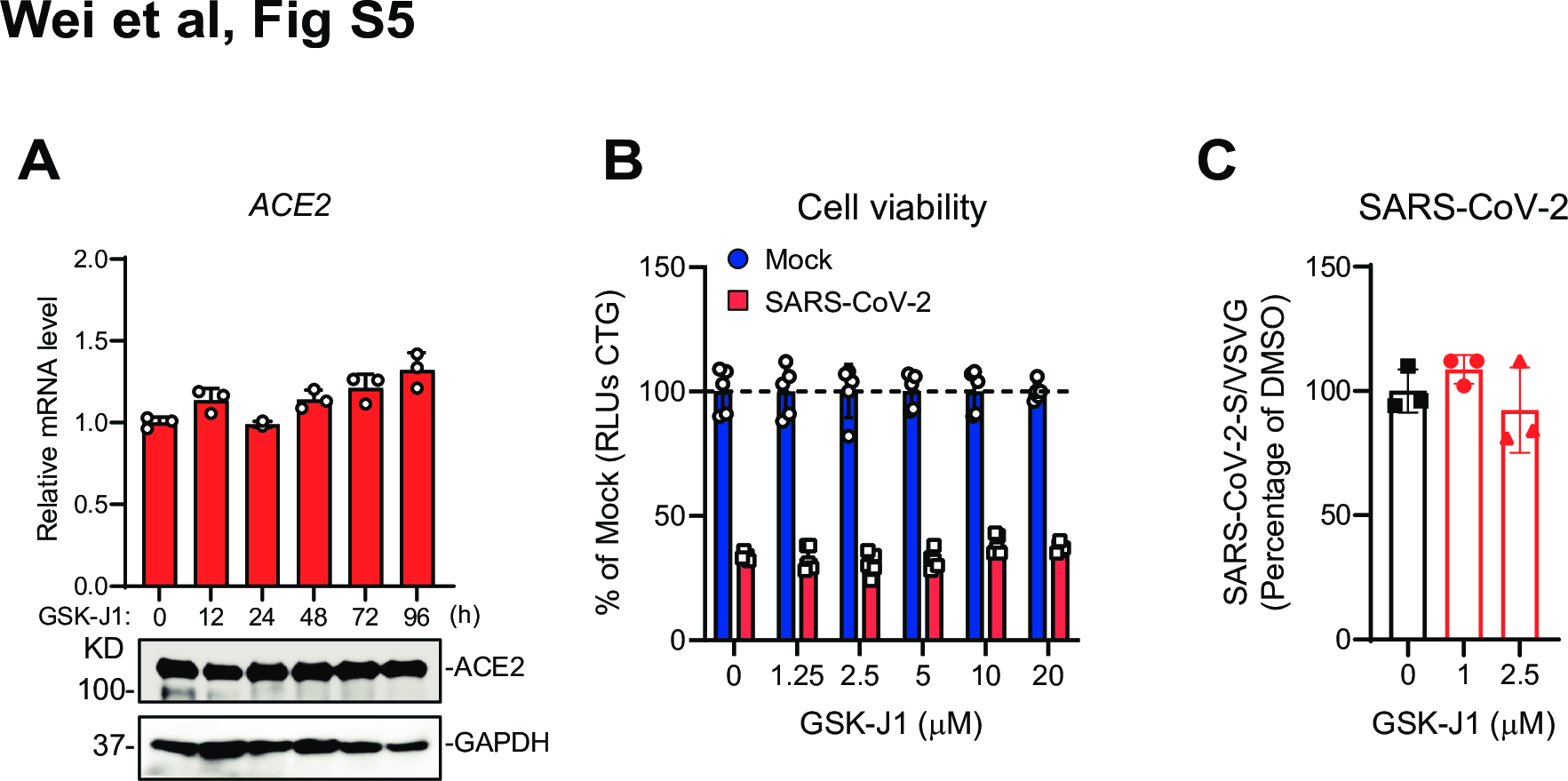

Supplement: S5 Fig — (A) Vero E6 cells were treated with 1.25 μM GSK-J1 for the indicated times, ACE2 mRNA and protein levels were measured by RT-qPCR and immunoblot, respectively. (B) Vero E6 cells were pretreated with GSK-J1 for 2 days and then infected with SARS-CoV-2 at an MOI of 0.2. Cell viability was measured at 3 dpi. (C) Vero E6 cells were pretreated with GSK-J1 for 2 days and then infected with VSV pseudovirus (VSVpp): VSV-G and SARS-CoV-2-S. Luciferase relative to the VSVpp-VSV-G control was measured 1 dpi. Data were analyzed by one-way ANOVA with Tukey’s multiple comparison test. Shown are mean ± SEM. *p < 0.05, **p < 0.01, ***p < 0.001. (TIF) [file ppat.1011351.s005.tif]
